# Equal versus equivalent access to the scientific literature

**DOI:** 10.1186/1742-4690-8-83

**Published:** 2011-10-21

**Authors:** Kuan-Teh Jeang

**Affiliations:** 1 The National Institutes of Health, Bethesda, MD, USA

## The concepts of equal versus equivalent access to the scientific literature are discussed

In 1954, the United States Supreme Court in a landmark decision of *Brown vs. Board of Education of Topeka, Kansas *sharply repudiated the "separate but equal" principle of public education. The Court concluded that racially segregated education is "inherently unequal". In scientific publishing today, there exist two segregated means of knowledge dissemination --- the subscription journals and the Open Access (OA) journals. For those who can pay, there is immediate access to scientific papers published in both subscription and OA journals; those who cannot pay can access only OA journals. The status quo is thus an "inherently unequal" playing field between the "haves" and the "have nots".

How unequal is the current situation? In an August 1, 2011 posting on the *Nature News *website, Richard Van Noorden reported that "the proportion of research papers freely available is slowly and steadily creeping upwards... in 2009, it's above 28%. (Some of this literature is not immediately available at the time that it is published, because of journal policies that impose embargo periods on when material can become free)". The good news is that approximately 30% of published papers can be accessed freely. The bad news is that 70% of published, publicly funded research remains off-limits to those who cannot pay.

Can equal access be had by the "haves" and the "have nots"? To the extent that the subscription and OA tracks will likely co-exist, the foreseeable future is a "separate and unequal" reality. Without equal access, the next best goal is perhaps to achieve equivalent access.

What is equivalent access? Imagine two very similar papers reaching essentially the same conclusions; one is published in a subscription journal and the other published in an OA journal. The paying reader can read both papers; the non-paying person can read only the OA paper. This is "unequal" access. However, if the OA paper sufficiently conveys the same information as the subscription paper, then it is possible that "equivalent" knowledge is conveyed to both the can-pay and cannot-pay audiences.

**Figure 1:**
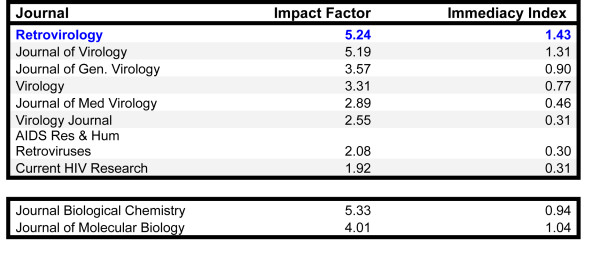
**Impact factor numbers from 2010 ISI-Thomson Reuters data that compare *Retrovirology *with nine other subscription journals**. Seven of the nine journals publish basic virological research papers. The *Journal of Biological Chemistry *and the *Journal of Molecular Biology *are included for comparison to two well-established journals that publish basic research papers in biochemistry and molecular biology.

The equivalent access concept works only if subscription and OA journals can attract and publish, in chronological proximity, similar articles of comparable quality and impact. Practically speaking, for this to occur, OA journals need to achieve quality metrics (e.g. Impact Factor numbers) that match their subscription counterparts. The *Retrovirology *experience suggests that such benchmark can be achieved (Figure 1).

Achieving qualitative parity will go a long way towards advancing equivalent access to important biological findings. One could raise the recent XMRV-Chronic Fatigue Syndrome controversy [1] as an example. A strongly credible case can be made that OA readers who read only *Retrovirology *papers [2-9] knowledgeably reached the equivalent scientific conclusion regarding this topic as those who read the subscription-based literature.

The quality of OA publishing will continue to improve. In 2012, *Cell *will launch a top tier OA publication, *Cell Reports*; and the Wellcome Trust/the Howard Hughes Medical Institute/the Max Planck Society will also start a similarly high profile OA journal. Because intelligence and ambition are distributed equally around the globe [10], freely available equivalent access to timely knowledge matters. "Separate but equivalent" may become the watchword of 21^st ^century publishing.
